# Ocular Imaging in Intraocular Lymphoma: A Review

**DOI:** 10.1111/ceo.70064

**Published:** 2026-01-19

**Authors:** Amin Ibrahim, Vishal B. Swaminathan, Wendy M. Smith, Lauren A. Dalvin

**Affiliations:** 1 Department of Ophthalmology, Mayo Clinic, Rochester, Minnesota, USA; 2 Department of Medical Oncology, Mayo Clinic, Rochester, Minnesota, USA

**Keywords:** biomarkers, intraocular lymphoma, multimodal imaging

## Abstract

Intraocular lymphomas, including vitreoretinal and choroidal lymphoma, can simulate the clinical presentation of other benign and malignant ocular diseases resulting in diagnostic delays. Multimodal imaging features can raise early clinical suspicion to support appropriate subspecialty referrals and treatment for patients affected by these conditions. This review synthesises current evidence on the diagnostic and prognostic value of characteristic imaging features of these rare malignancies. Findings are reviewed based on imaging modality, including fundus photography, optical coherence tomography, fundus autofluorescence, fluorescein angiography, indocyanine green angiography, optical coherence tomography angiography, and ultrasound. We emphasise discriminative biomarkers that heighten suspicion for either vitreoretinal or choroidal lymphoma, as well as key findings to discriminate between lymphoma and alternative diagnoses. We further describe longitudinal changes in multimodal imaging features that can facilitate tracking disease progression or treatment response. By consolidating modality-specific findings, this review aims to facilitate early referral and accurate diagnosis of these rare malignancies.

## 1 | Introduction

Intraocular lymphoma is a non-specific term used to describe malignant lymphoproliferative disease occurring in the eye [1]. Intraocular lymphomas have both primary forms, arising from within the eye itself, and secondary forms, resulting from the spread of systemic lymphoma [1]. They can also be anatomically categorised as either vitreoretinal lymphoma or uveal lymphoma, the latter of which most commonly affects the choroid (choroidal lymphoma) [1].

Although rare, intraocular lymphoma has received growing attention in recent years. A US study looking at ocular and orbital lymphomas between 1995 and 2018 reported an age-adjusted incidence rate of 0.245 cases per million for intraocular lymphoma [2]. The incidence rate has fluctuated over the decades, with the trend recently decreasing [2]. Most patients (58.4%) were between the ages of 60 and 79, with females being affected slightly more than males [2]. Long-term survival outcomes show a positive trend, with the 5-year cumulative survival estimated at 70.6% [2].

This review focuses on the evolving role of ocular imaging modalities in the diagnosis and monitoring of intraocular lymphomas, including vitreoretinal and choroidal lymphoma (Table 1). Understanding the imaging features of these rare malignancies can aid in earlier detection, more accurate diagnosis, and improved patient outcomes.

## 2 | Vitreoretinal Lymphoma

Vitreoretinal lymphoma (VRL) arises in the vitreous and/or retinal compartments of the eye. Most primary vitreoretinal lymphomas (PVRL) are non-Hodgkin diffuse large B-cell lymphomas, but rare cases of T-cell lymphoma have been described [3–5]. PVRL is considered a subset of primary central nervous system lymphoma (PCNSL). The percentage of patients who had PVRL and later develop central nervous system involvement (CNSL) varies from 36% to 85% [6–8], while the percentage of patients with PCNSL who develop intraocular involvement ranges from 15% to 25% [7, 8]. Diagnosing VRL can be challenging, with the reported misdiagnosis rate around 64% [9]. VRL often mimics inflammatory uveitis, earning it its label as a masquerade syndrome [10, 11]. Consequently, patients misdiagnosed and initially treated with corticosteroids often face delayed diagnosis and referral times [12]. Cytopathology remains the gold standard for diagnosing VRL, although sensitivity varies and may be higher in tertiary referral centres with experience handling delicate vitrectomy specimens. To prompt early referral, advancements in multimodal imaging techniques, including fundus photography, optical coherence tomography (OCT), fundus autofluorescence (FAF), fluorescein angiography (FA)/indocyanine green angiography (ICGA), OCT angiography (OCTA), and ultrasound, have proved useful to assist in early detection and monitoring of VRL [13–15]. Herein, we describe key multimodal imaging features that can help suggest a VRL diagnosis.

### 2.1 | Fundus Photography in Vitreoretinal Lymphoma

Fundus photography may be used for serial comparisons to monitor size and colour of lesions. Images limited to the posterior pole in eyes with VRL are far from ideal, as VRL involvement often extends beyond the posterior pole and midperipheral retina [16]. Ultra-widefield (UWF) fundus photography addresses these limitations. In fact, a series examining 41 eyes using both UWF and standard 30° fundus photography found that the former detected additional abnormalities in 47% of eyes [17]. Limitations to UWF fundus photography can include less attention to fine macular details and distortion of the periphery of the image [16]. Features of VRL seen on fundus photography can include vitreous cellular infiltration, intraretinal, subretinal, or sub-retinal pigment epithelium (RPE) lesions, optic nerve head oedema, and retinal detachment [17].

**FIGURE 1 |:**
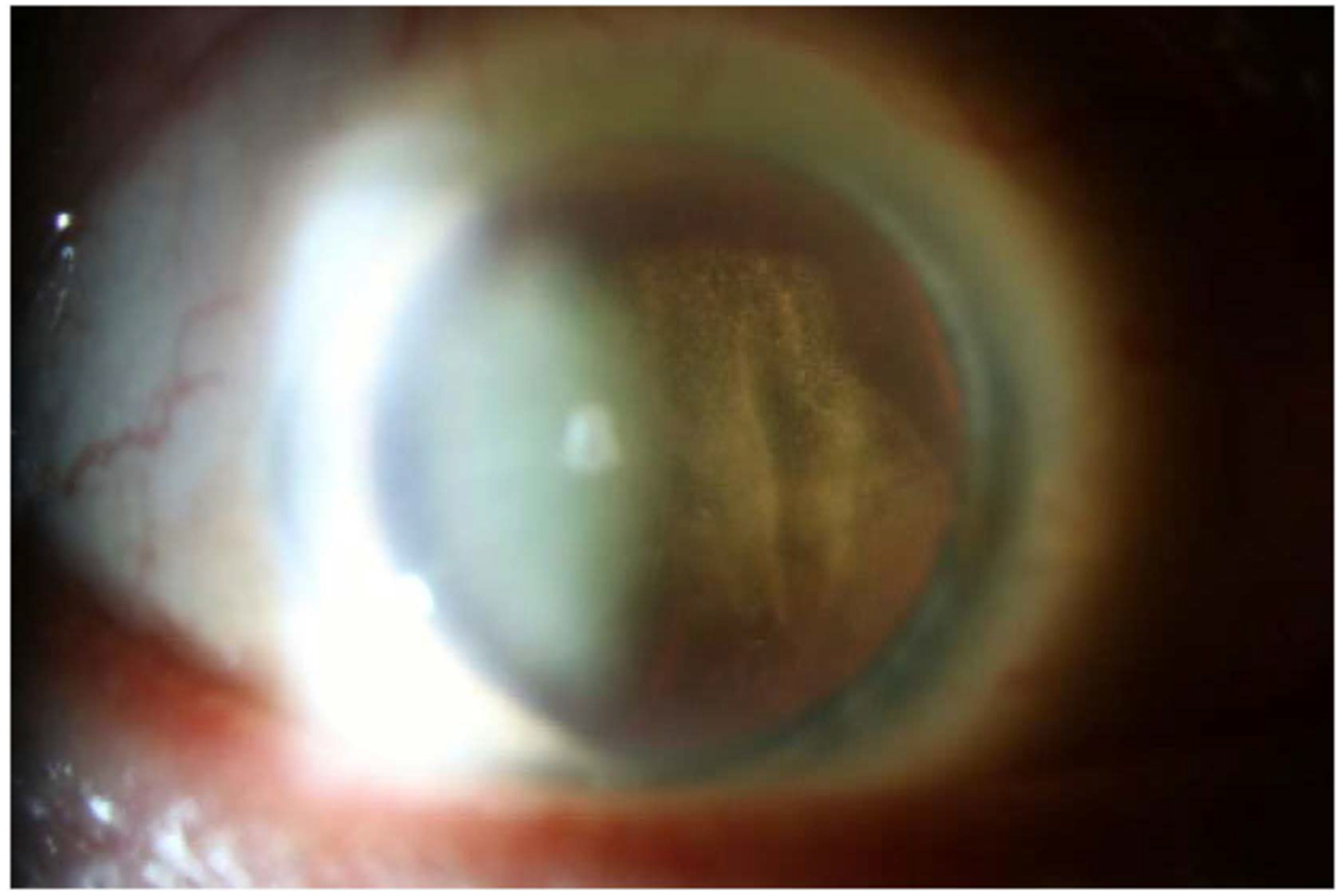
Slit-lamp photograph of vitreous cell in a patient with vitreoretinal lymphoma. Vitreous cell is a common feature in vitreoretinal lymphoma, which can be well-visualised with careful slit lamp examination.

The presence of vitreous cellular infiltration is a classic feature of VRL and several patterns have been described [18]. The aurora borealis pattern is the most specific for VRL, described as linear opacities with a myriad of cells aligned along a radial texture of vitreous fibrils scattering the light beam and thus creating an aurora borealis appearance [18]. When seen, this pattern should raise suspicion for lymphoma over uveitis. Another less frequent but distinctive presentation is a string of pearls pattern described as fine fibrils connecting bunches of inflammatory material; however, this is a nonspecific feature that may be shared with sarcoidosis or infectious uveitis [18]. Nonspecific vitreous strands and pockets have also been described [18]. While such features may not be well captured on fundus photography, merely contributing to reduced image quality for direct visualisation of the fundus, vitreous infiltration of VRL can be well captured using post-dilation slit lamp photography (Figure 1).

Quantitatively, the aurora borealis pattern has been reported in roughly one-third of VRL cases, whereas the nonspecific pattern was slightly more common [18, 19]. Vitreous infiltration is an important clinical indicator as it may be the only sign of posterior segment involvement in 40% of eyes, with about 90% of eyes having one of the three patterns discussed [18, 19]. Notably, visual acuity may remain unexpectedly good despite the presence of clinically significant vitreous media opacities [18]. Such a presentation should prompt a high index of suspicion for VRL over uveitides which more commonly cause macular edema and significant vision loss [20].

**FIGURE 2 |:**
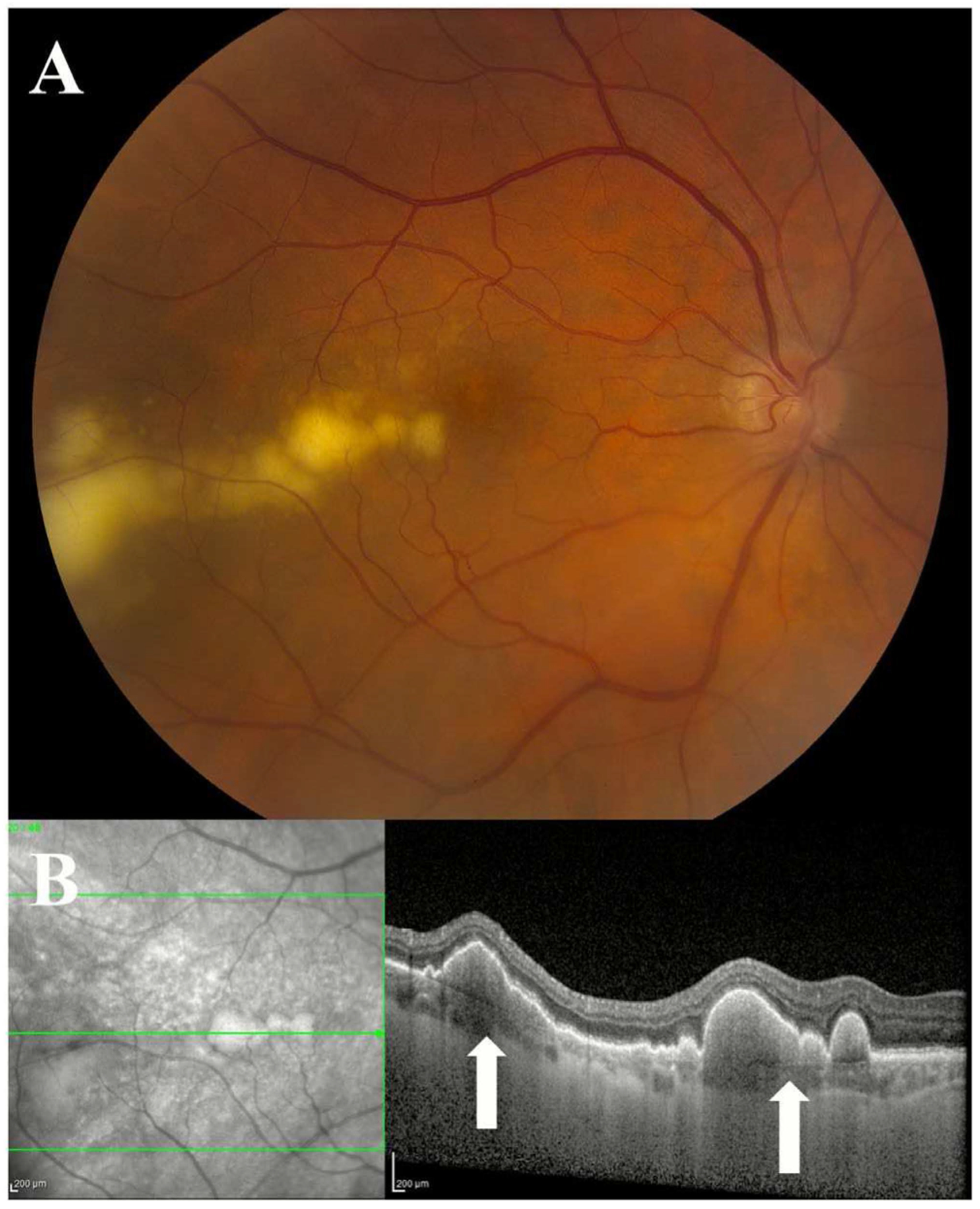
Colour fundus photograph of sub-retinal pigment epithelium (RPE) deposits in vitreoretinal lymphoma. (A) Sub-RPE deposits, visualised as yellow lesions deep to the retina, are a characteristic finding in patients with vitreoretinal lymphoma. (B) The lesions can be seen on OCT as hyperreflective deposits beneath the RPE. Focal sub-RPE deposits in VRL can be a helpful feature to distinguish VRL from inflammatory uveitis.

**FIGURE 3 |:**
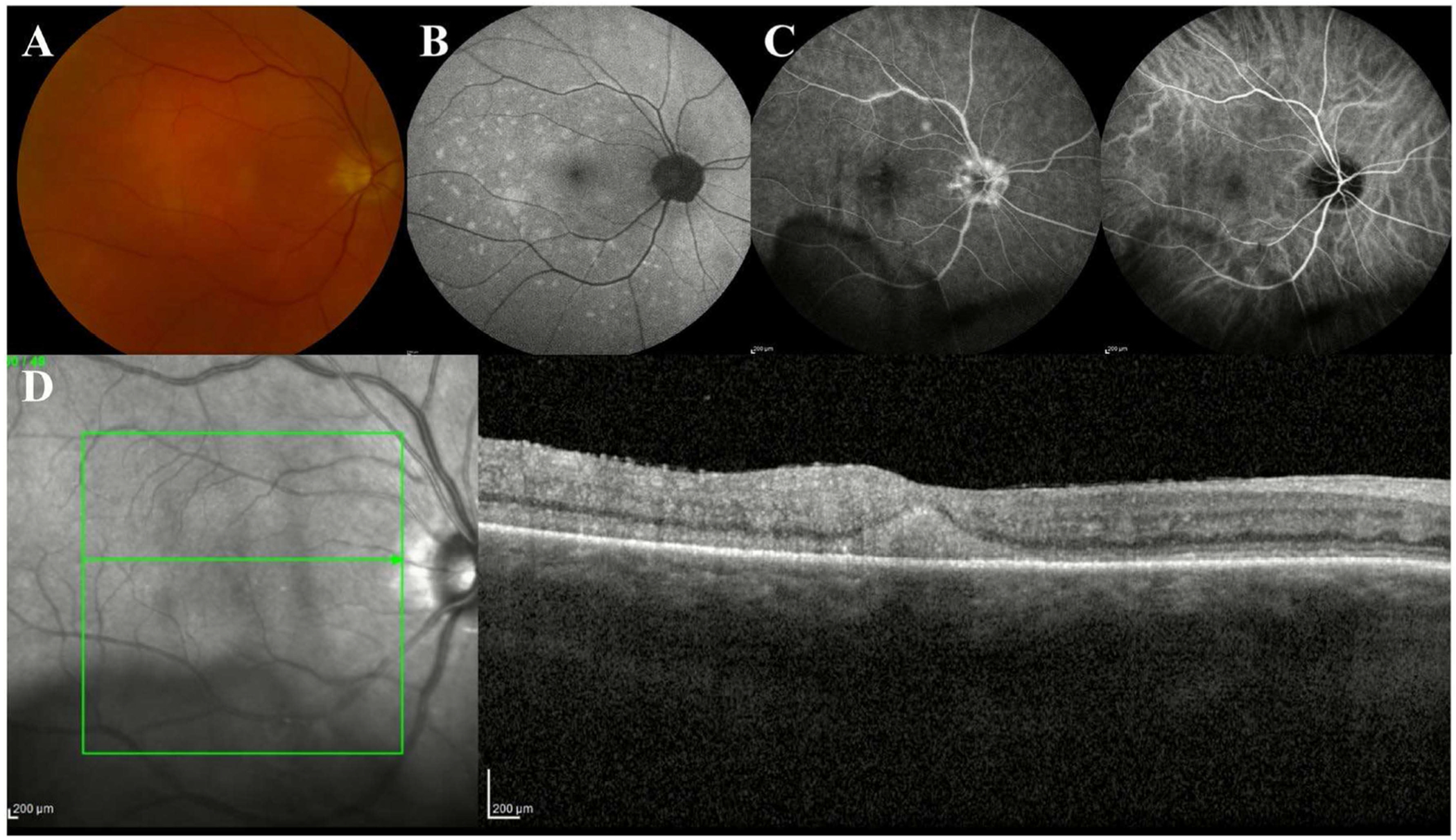
Multimodal imaging in vitreoretinal lymphoma (VRL). (A) Fundus photograph reveals deep, cream-coloured lesions in the posterior pole with (B) scattered hyperautofluorescent foci on fundus autofluorescence imaging. (C) Fluorescein angiography shows hyperfluorescent foci in the posterior pole and overlying the optic nerve, suggesting infiltration with some hypofluorescent foci seen on indocyanine green angiography. (D) Pre-retinal deposits are visualised on OCT overlying the internal limiting membrane with scattered hyperreflective intraretinal infiltration throughout the macula. Subretinal infiltration appears as a hyperreflective deposit beneath the retina with fuzzy borders of the outer retinal layers.

Retinal infiltration can be seen on fundus photography as creamy, white or yellow retinal thickening. Subretinal or sub-RPE invasion can be seen as creamy subretinal or sub-RPE lesions that may or may not present with haemorrhages (Figures 2A and 3A). A rare but classic presentation, multifocal subretinal lesions may coalesce over time and appear as a leopard spot pattern [17]. Subretinal lesions have been reported in up to 81% of eyes with VRL [21], whereas the leopard spot pattern is seen in only 9%–21% [21, 22].

### 2.2 | Optical Coherence Tomography in Vitreoretinal Lymphoma

Optical coherence tomography (OCT) is non-invasive, rapid, reproducible, readily available and can help raise suspicion for a VRL diagnosis without any risk to the eye (Table 2) [23, 24]. The importance of this modality was underscored by a survey of uveitis specialists and ocular oncologists, in which 100% of respondents agreed that OCT was a required diagnostic procedure for identifying VRL [14]. A growing body of observational cohort studies demonstrates that, beyond its diagnostic use, OCT is also a valuable tool for longitudinally assessing disease activity and response to treatment, as it can detect significant changes such as resolution of vitreous opacities, intraretinal infiltration, subretinal infiltration, and normalisation of retinal architecture, which correlate with tumour regression [16, 25–30]. OCT has evolved into a critical tool not only for imaging but also for understanding and managing disease behaviour [27, 31, 32].

#### 2.2.1 | Classic Findings on OCT

##### 2.2.1.1 | Vitreous.

Vitreous cells appear as hyperreflective foci in the posterior vitreous on OCT [16, 26]. They are nearly ubiquitous, reported in up to 100% of eyes with VRL, but can also be seen in eyes with uveitis [19, 26, 27, 30]. As one of the first clues that raise suspicion of VRL, vitreous cells demonstrate a sensitivity of 93% [16, 33]. The resolution of vitreous cells following intravitreal methotrexate therapy has been well documented as a sign of successful disease response [30].

**FIGURE 4 |:**
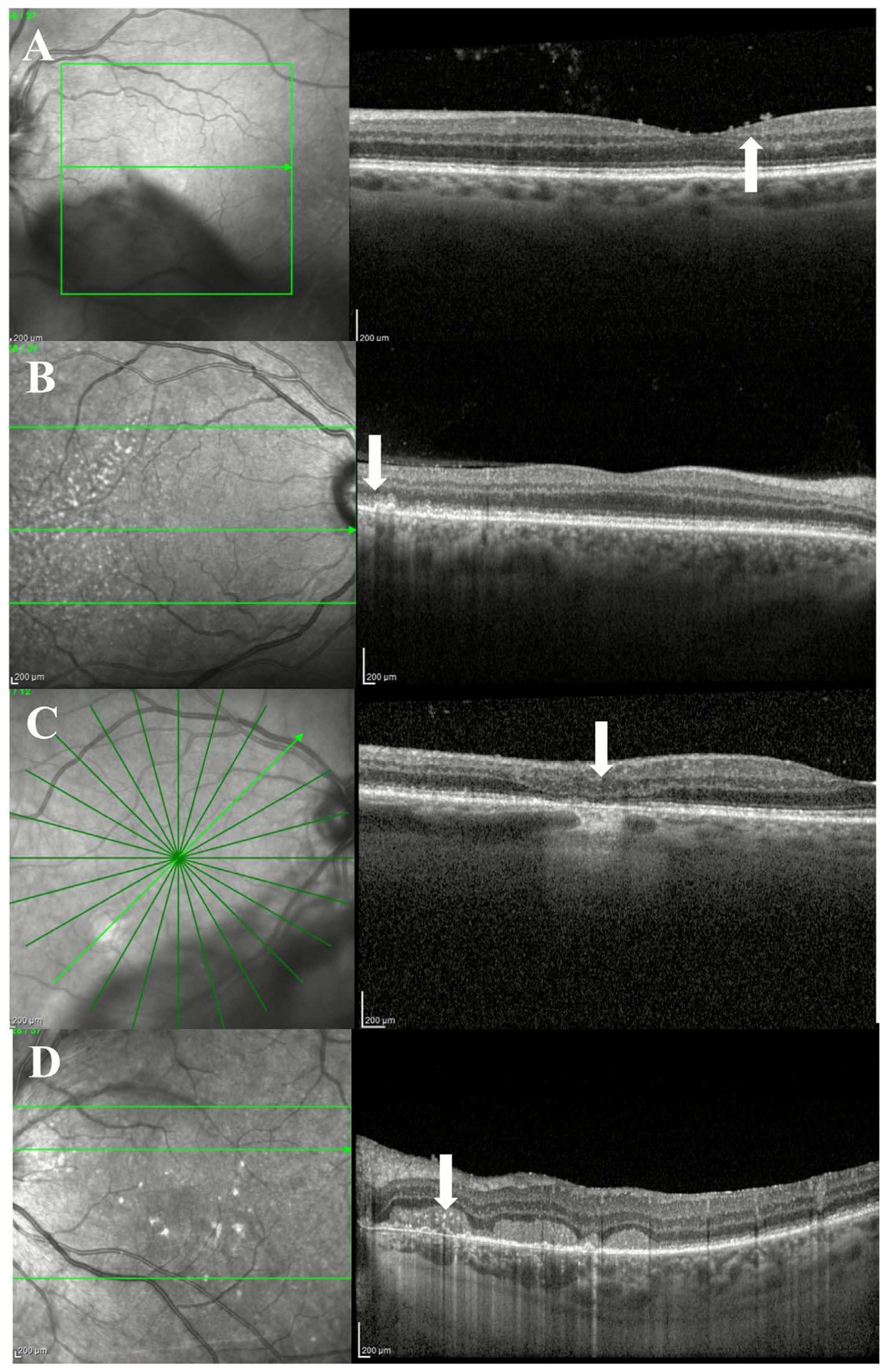
OCT findings in vitreoretinal lymphoma (VRL). (A) Pre-retinal deposits appear as hyperreflective material resting on the inner retinal layer. (B) Outer retinal changes may appear as fuzzy borders with RPE involvement, in this case with nodularity. (C) Outer retinal atrophy with ellipsoid zone disruption can represent irreversible damage to the photoreceptor outer segment. (D) Subretinal infiltration appears as hyperreflective deposits beneath the retina, which can take on a band-like configuration (arrow).

Preretinal deposits are visualised as hyperreflective material resting on the inner retinal layer (Figure 3D) [27]. While they are less common, their presence carries a strong diagnostic weight as they are considered a highly specific feature for VRL (92.5% specificity) (Figure 4A) [33]. Their reported prevalence ranges from 13% to 44.4% [24, 25, 27, 33, 34]. The prevalence of preretinal deposits did not change at initial presentation compared with maximum progression, recurrence, or regression [27]. All preretinal deposits disappeared following pars plana vitrectomy in another cohort, however [25]. Their presence on OCT may be overlooked in eyes with dense vitreous opacities, potentially leading to under-recognition of this otherwise specific sign [34].

##### 2.2.1.2 | Retina

###### 2.2.1.2.1 | Intraretinal Infiltration.

While intraretinal infiltration is less common overall in VRL (observed in only 7% of eyes in the largest cohort to date of 182 eyes), its presence can be highly specific [21]. Intraretinal infiltration can present with several distinct patterns on OCT, including diffuse intraretinal deposits, focal round lesions, and vertical hyperreflective lesions, which may have some overlapping features (Figure 3D) [27, 28, 35].

Diffuse intraretinal deposits are a highly specific (100% specificity) pattern of retinal involvement in VRL, visualised as hyperreflective material manifesting as diffuse retinal thickening accompanied by disorganisation of the retinal layers. This feature can help point towards a VRL diagnosis over uveitis [27, 33]. Focal intraretinal deposits are another pattern, visualised as small, isolated hyperreflective lesions in the outer retinal layer [27]. A further distinct pattern includes focal round lesions, visualised as hyperreflective infiltrates within the neural retina that are comparable in size or larger than vertical hyperreflective lesions [28].

Intraretinal vertical hyperreflective lesions (VHRL) are a highly suggestive feature of VRL [33, 35]. In a series of 7 patients with VRL, VHRLs were described as affecting all layers of the neural retina, exhibiting moderate or high reflectivity, preceding the development of sub-RPE deposits, and often localising around second- and third-order retinal vessels [35]. VHRLs can be classified as complete (extending from RPE to the ganglion cell layer), incomplete (extending from the RPE to the neuroretina), or mixed (both of the aforementioned) [33].

The vertical complete and the vertical mixed patterns demonstrate a specificity of 100% for VRL and can help differentiate it from acute syphilitic posterior placoid chorioretinitis, chronic stage sympathetic ophthalmia, or idiopathic multifocal choroiditis, whereas the vertical incomplete pattern may also be seen in these uveitic entities and is therefore nonspecific [33]. VHRLs are reported to resolve with minimal to no scarring post-chemotherapy [29, 35].

Successful treatment should lead to resolution of intraretinal infiltrates; thus, they can be used to monitor therapeutic response. In a series of 111 eyes of 58 patients with PVRL, between initial presentation and regression, all intraretinal infiltrates resolved with intravitreal methotrexate (39.6% vs. 0% (vs), *p* < 0.001) [30]. Conversely, intraretinal infiltrates decreased but did not completely disappear following intravitreal rituximab (55.6% vs 16.7%, *p* = 0.002) [29].

The development of intraretinal deposits is a late finding that should raise the possibility of disease progression or recurrence [28, 30]. In fact, in a series of 55 eyes of 32 patients with VRL, compared to initial presentation both diffuse and focal intraretinal lesions were seen more frequently at the time of tumour progression (17% vs. 50% of eyes) or recurrence (7% vs. 47% of eyes) [27]. Furthermore, intraretinal infiltration has been associated with subsequent development of CNS manifestations, with an odds ratio of 4.3 [36].

Retinitis-like lesions represent a distinctive feature of VRL mimicking infectious retinitis; they appear as massive retinal thickening without significant tissue loss, resulting in multiple peaks and slopes on OCT, described as the ‘rounded roof’ appearance [37]. To better define this presentation, the term ‘pseudonecrotic retinal lesions’ (PRLs) has been introduced, highlighting the relative preservation of retinal tissue in contrast to the tissue destruction observed in infectious retinitis [38]. Clinically, PRLs are associated with a more severe presentation and a grim prognosis [38]. In a series of 67 eyes of 40 patients with biopsy-proven VRL, eyes with PRLs had worse best-corrected visual acuity (BCVA) and more severe clinical presentation than their non-pseudonecrotic counterparts, with features including optic disc swelling, retinal vasculitis, retinal haemorrhage, and retinal detachment [38]. Consequently, PRLs are also associated with considerably worse final visual acuity outcomes, with nearly half of affected eyes achieving a final BCVA of hand motions or worse [38].

###### 2.2.1.2.2 | Outer Retina.

The outer retina is more susceptible to involvement in VRL than the inner retina [25]. Several outer retinal abnormalities have been described in VRL, including outer retina (OR) fuzzy borders, outer retinal atrophy, disruption of the outer retina, and ellipsoid zone (EZ) disruption. Importantly, restoration of normal or near normal retinal architecture can be achieved following VRL regression if treated early [25, 29].

OR fuzzy borders, a common but nonspecific finding in VRL, correlate with disease activity in VRL but also with other retinal and choroidal disorders [29, 34]. Fuzzy borders, visualised as blurring of the physiologic external retinal boundaries including the inner segment/outer segment junction (IS/OS), RPE, and external limiting membrane (Figure 4B) [26, 29], have been reported in 45.5% to 73.7% of eyes with VRL [25, 26, 29, 34].

Outer retinal atrophy can be a useful clue when differentiating VRL from uveitis. It occurs more often in VRL, affecting 22% of eyes with VRL compared with 3% of eyes with inflammatory uveitis (*p* < 0.001). Although predictive of VRL on univariate analysis, it was not on multivariate analysis, highlighting the need for interpretation within broader clinical context [24].

EZ disruption is a frequent OCT finding in VRL, with a reported frequency of 52.3% to 73.3% of affected eyes [16, 30, 34]. In a series of 111 eyes with biopsy-proven PVRL, while features such as vitreous cells, intraretinal infiltrates, and pigment epithelium detachments (PEDs) completely resolved, EZ disruption was the most frequent morphologic characteristic at the last study visit despite treatment with a mean of 12 intravitreal methotrexate injections [30]. Persistence of EZ disruption has been hypothesised to reflect irreversible damage to the photoreceptor outer segment resulting from lymphomatous infiltration of the RPE (Figure 4C) [26, 30].

Other outer retinal abnormalities include hyperreflective retinal dots (HRD), which appear as focal hyperreflective lesions predominantly found in the outer retinal layers [29]. In a series of 18 eyes of 9 patients, HRDs were the most common feature in treatment-naïve eyes, but they aren’t specific for VRL [29]. HRDs have a distinct appearance from other intraretinal lesions, which have poorly demarcated borders, lower reflectivity, and larger size [29]. Cloudy vitelliform submaculopathy is a less common feature described as a macular detachment caused by transient hyperreflective debris above an irregularly rippled or thick RPE layer [21, 39]. The frequency of cloudy vitelliform submaculopathy in VRL ranges from 6% to 9% [21, 40].

###### 2.2.1.2.3 | Other Retinal Features.

Cystoid macular edema (CME) is classically considered uncommon in VRL, with its absence traditionally serving as a useful clinical clue distinguishing VRL from inflammatory etiologies, especially in cases of marked vitreous activity. While several studies have reported CME as a secondary OCT finding in VRL, with a frequency ranging from 5.6% to 17% [16, 17, 20, 24, 29], it is important to note that these reported rates are often influenced by potential confounding factors such as previous surgery, chemotherapy, or radiation. In a series of 15 eyes with VRL specifically designed to determine the occurrence of macular edema (ME) in VRL, ME was concluded to be a rare finding as an initial presentation of VRL and more likely a consequence of prior interventions [20]. After the exclusion of eyes with such confounders, the rate of ME was 0% [20]. Similarly, a comparative series of 95 eyes with VRL and 86 eyes with inflammatory uveitis found CME to occur more frequently in uveitis than in VRL (36.7% vs. 11.7%, respectively) [24]. Although uncommon, it is important to remember that CME does not exclude a VRL diagnosis. Clinicians should maintain appropriate suspicion if the clinical context is suggestive of VRL, even if CME is present.

###### 2.2.1.2.4 | Subretinal Space.

The presence of infiltration within the subretinal space can be seen in both VRL and certain forms of uveitis such as acute syphilitic posterior placoid chorioretinitis, chronic stage sympathetic ophthalmia, and idiopathic multifocal choroiditis [33]. Thus, interpretation of this feature should be made based on clinical context. In VRL, homogeneous hyperreflective infiltrates within the subretinal and sub-RPE spaces are key findings that can help differentiate VRL from infectious retinitis [37]. Two main morphologically distinct patterns of lymphomatous subretinal infiltration have been described: hyperreflective discrete nodules (also described as focal or dots) and hyperreflective confluent bands (Figure 4D) [23]. The latter has a specificity of 90% for VRL and should be considered highly suggestive of this diagnosis [23, 33]. Subretinal infiltrates have been documented to decrease or even completely resolve following intravitreal methotrexate, reinforcing the practical use of OCT as a useful non-invasive tool for monitoring treatment response [26, 30].

However, not all hyperreflective subretinal lesions disappear completely. In one series examining 19 eyes with VRL, hyperreflective subretinal dots, located between the RPE and the ellipsoid zone and attached to the RPE, persisted in a subset of eyes following tumour eradication and were presumed to represent non-absorbable cellular debris rather than active disease [25]. Importantly, these persistent dots were associated with structural and functional photoreceptor damage. In a later series of 11 eyes with VRL assessed using OCT and electroretinography (ERG), all eyes with significant attenuation in both dark- and light-adapted a-waves on ERG exhibited hyperreflective subretinal dots, while eyes demonstrating a normal ERG had a normal EZ and lacked hyperreflective sub-retinal dots [34]. These findings support a sequence in which lymphoma cells first infiltrate the sub-RPE/subretinal space, disrupt the RPE, and subsequently induce both structural (EZ disruption) and functional (a-wave attenuation) photoreceptor damage, which may persist despite successful tumour eradication [34].

Although subretinal fluid may reflect a chronic course of VRL, the presence of subretinal or subfoveal fluid often serves as helpful clues in pointing towards a diagnosis of uveitis, specifically intermediate uveitis or sarcoid posterior uveitis [24, 30]. In a series of 95 eyes with VRL compared to 86 eyes with inflammatory uveitis, subretinal fluid occurred more frequently in eyes with uveitis (6.4% vs. 16.3%) [24].

###### 2.2.1.2.5 | RPE.

Several RPE structural abnormalities have been documented in eyes with VRL including undulations (rippling), nodularity, PEDs, and even rips [16, 17, 23–26, 30]. Among these, diffuse RPE elevations are a relatively sensitive feature for VRL (56.5% sensitivity) whereas confluent RPE detachments are highly suggestive of VRL [33]. RPE changes can also be commonly seen in certain uveitic entities (intermediate uveitis and biopsy-confirmed sarcoid posterior uveitis) but are typically absent in others (acute syphilitic posterior placoid chorioretinitis, chronic stage sympathetic ophthalmia, and idiopathic multifocal choroiditis); thus, their presence should be interpreted within the broader clinical context [24, 38].

Beyond their diagnostic value, certain morphologic RPE features offer clues regarding disease severity and prognosis. In a series of 43 eyes of 23 patients with biopsy-proven PVRL, RPE nodularity was associated with macular granularity over leopard spotting on FAF and may persist with EZ loss after treatment of eyes with PED, leading to reduced visual acuity [17].

Population-based differences may also refine interpretation. In a series of 134 eyes with biopsy-proven PVRL comparing American and Asian populations, RPE undulations appeared more frequently in the Asian population (61% vs. 16%), and their presence was associated with partial rather than complete regression after treatment (odds ratio of 4.3) [36]. This finding suggests RPE undulation could be a marker of more refractory local disease, though it was not linked to CNS progression or occurrence [36]. Reduction in RPE abnormalities following intravitreal methotrexate has been well-documented [30].

###### 2.2.1.2.6 | Sub-RPE.

**FIGURE 5 |:**
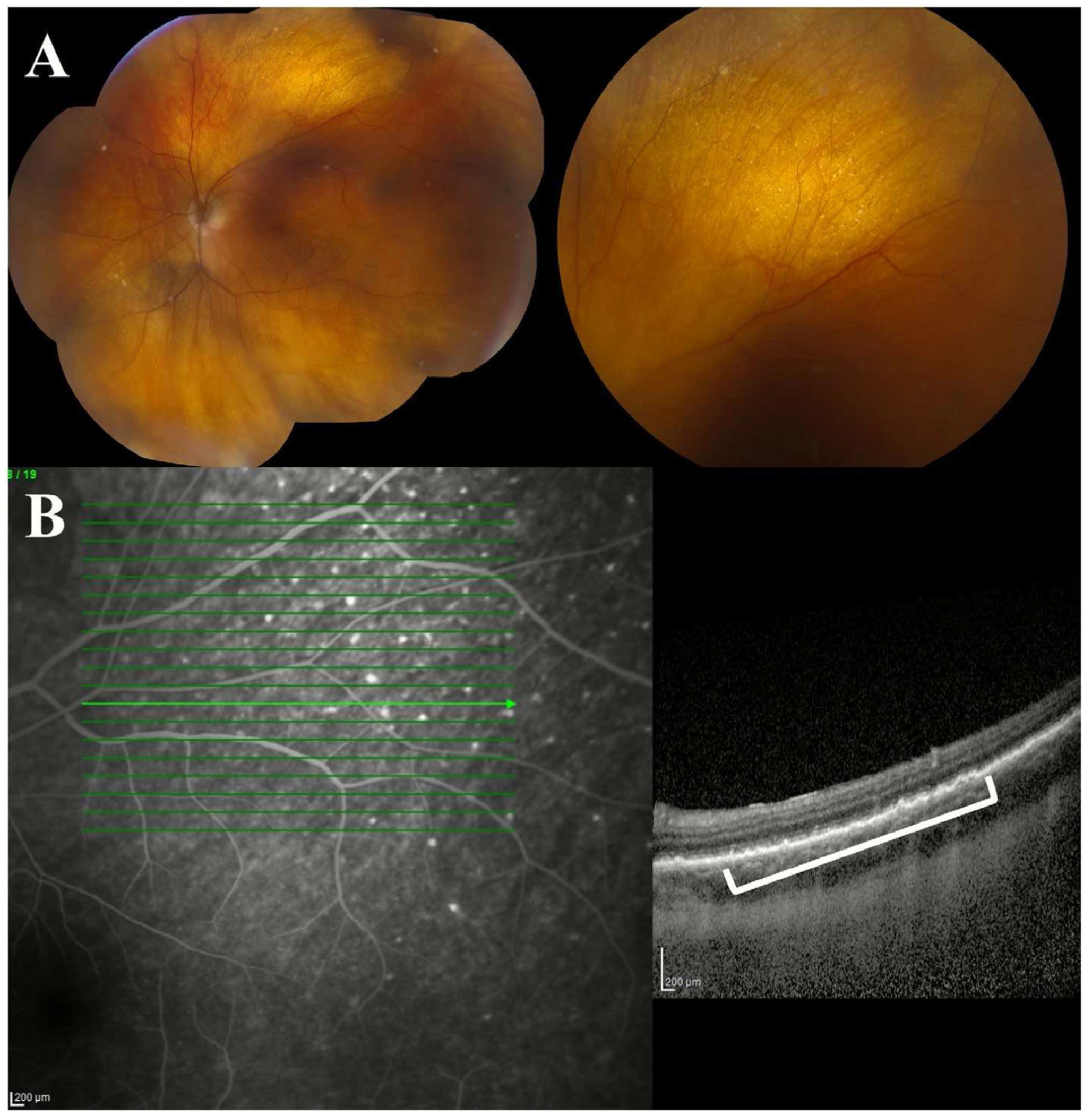
Sub-Retinal Pigment Epithelium (RPE) Hyperreflective Deposits in Vitreoretinal Lymphoma. (A) Fundus photographs may reveal localised lesions that appear as small, cream-coloured foci. (B) Lesions may correspond to sub-RPE hyperreflective foci on optical coherence tomography.

Sub-RPE lesions are visualised as hyperreflective deposits between the RPE layer and Bruch’s membrane, and they can present with several distinct patterns on OCT, including focal (solitary dome-shaped nodules), confluent (multiple coalescing dome-shaped nodular deposits), and diffuse minimally elevated (narrow diffuse band-like deposits) (Figures 2B and 5B) [24, 27, 40]. Clinically, these sub-RPE lesions may correspond to small cream-coloured foci on fundus photography (Figure 5A). In a series of 182 eyes with VRL, sub-RPE infiltration was seen in 91% of eyes, and sub-RPE lesions with or without subretinal lesions were the most common baseline presentation, found in 85% of eyes [21]. This pre-dominance of sub-RPE abnormalities led to the hypothesis of a choroidal vascular origin of VRL [21]. The reported frequency of sub-RPE abnormalities, however, varies greatly from 9.4% to 100% [16,19, 23, 27, 37, 41].

Diagnostically, the presence of sub-RPE deposits should raise suspicion for VRL, but this feature is not pathognomonic and can also occur in certain forms of uveitis. Sub-RPE deposits are typically absent in intermediate uveitis and sarcoid posterior uveitis but may be present in acute syphilitic posterior placoid chorioretinitis, chronic stage sympathetic ophthalmia, and idiopathic multifocal choroiditis at rates similar to VRL [24, 33].

Prognostically, in a series of 95 patients with VRL, sub-RPE infiltration was associated with a shorter mean time to death (46 vs. 76 months) on multivariate analysis but not competing risk analysis [32]. Additionally, in a study of 55 eyes of 32 patients with VRL, the prevalence of sub-RPE deposits did not change at initial presentation compared with maximum progression or recurrence, but was lower at regression [27].

###### 2.2.1.2.7 | Choroid.

Inner choroidal infiltration, visualised as hyperreflective pinpoint lesions, may occur in VRL, but their presence is a useful clinical clue favouring a uveitis diagnosis. In a series of 45 eyes with VRL and 40 eyes with infectious or non-infectious uveitis, inner choroidal infiltrations were detected on OCT in 97.5% of eyes with uveitis compared with only 44.4% of eyes with VRL [33].

### 2.3 | Fundus Autofluorescence in Vitreoretinal Lymphoma

Fundus autofluorescence (FAF) allows for the functional assessment of RPE integrity through detection of lipofuscin distribution. In eyes with VRL, hyperautofluorescence generally corresponds to active lesions, hypothesised to result from altered RPE metabolism caused by underlying lymphomatous infiltrates in the sub-RPE space (Figure 3B) [16, 42]. Conversely, hypoautofluorescence may represent areas of RPE atrophy or signal blockage by VRL cells at the RPE level [22].

**FIGURE 6 |:**
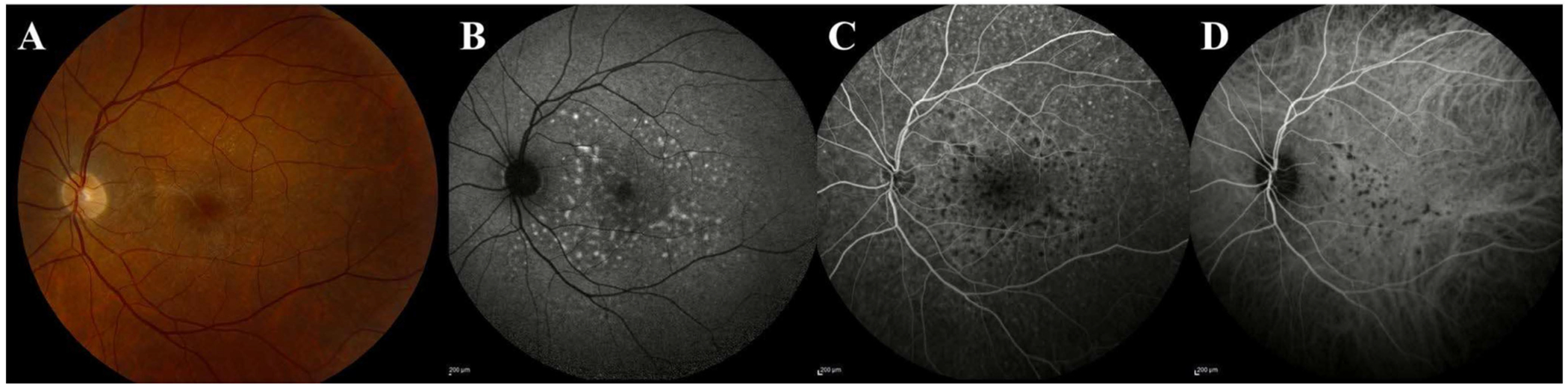
Multimodal image findings of vitreoretinal lymphoma (VRL). (A) Colour fundus photograph in VRL illustrating multifocal, deep creamy lesions that correspond to (B) hyperautofluorescent foci on fundus autofluorescence imaging, taking on a leopard-spot or granular pattern. (C) Fluorescein and (D) indocyanine green angiography in VRL can highlight hypofluorescent foci of lymphoma infiltration, with additional hyperfluorescent foci in the periphery of the fluorescein image.

Several distinct patterns of VRL involvement have been reported on FAF. Most notable, a granular pattern reported in up to 100% of eyes with FAF abnormalities [17, 42], may be visualised as alternating hyper- and hypoautofluorescent spots measuring 50–150 μm that are distributed across the posterior pole and peripheral retina (Figure 6B) [42]. Another related but distinct pattern is the leopard spot appearance, characterised by multiple hypoautofluorescent spots with hyperautofluorescent rims, with a reported frequency of 34%–56% [19, 42, 43]. This finding is associated with other ocular disorders, both malignant and benign; thus, its presence necessitates ruling out any possible underlying malignancies [19]. Other findings include focal hyperautofluorescent spots that may coalesce into a reticular pattern and blockage by mass lesions visualised as hypoautofluorescence [22, 42].

In a series of 16 eyes with PVRL, the presence of abnormal findings on FAF demonstrated a moderate sensitivity of 68.75% and high specificity of 100% in distinguishing active from inactive disease [42]. Thus, while the presence of FAF abnormalities may indicate active disease, their absence does not rule out PVRL presence or recurrence [42]. Conversely, a case was reported where hyperautofluorescent lesions were present despite inactive disease [17]. These observations highlight the importance of interpreting FAF findings in conjunction with other imaging modalities when assessing disease activity.

Hyperautofluorescent lesions may resolve with successful treatment. In a series of 15 treatment-naïve eyes with VRL receiving a mean of 5.7% intravitreal rituximab injections, hyperautofluorescence, corresponding to active lesions, diminished with each treatment cycle and was eventually replaced by hypoautofluorescence with or without a hyperautofluorescent rim [16]. In eyes with RPE rips, the rim corresponded to scrolled RPE [16].

FAF findings correlate with several features seen on other imaging modalities. The granular pattern on FAF is reversed on FA, with hyperautofluorescent spots corresponding to hypofluorescent spots; these spots are further correlated with nodular hyperreflective spots within the RPE on OCT [17, 42].

### 2.4 | Fluorescein and Indocyanine Green Angiography in Vitreoretinal Lymphoma

#### 2.4.1 | Fluorescein Angiography

Fluorescein angiography (FA) reveals a wide spectrum of vascular and RPE abnormalities associated with VRL. While a normal FA in VRL is possible, it is uncommon, with abnormalities present in up to 95% of eyes [17, 19, 22, 42]. The most common abnormalities on FA include pigment mottling, scleral staining, window defects, and diffuse vascular leakage. However, there is a paucity of published studies directly comparing FA findings in VRL with those of other differential diagnoses, limiting conclusions about their diagnostic value.

Breakdown of the blood-retinal barrier is a common angiographic feature of VRL [22]. Diffuse vascular leakage, found in 77% of eyes, presents in several distinct patterns depending on the disease stage [17]. In eyes without clinically apparent sub-retinal lesions, leakage primarily originates from medium- and small-calibre vessels, suggesting this as an early sign of lymphomatous involvement [17]. This finding typically resolves following treatment and serves as a marker of disease recurrence [17]. Conversely, in eyes with established subretinal lesions, a pattern of diffuse leakage from small vessels surrounding the infiltrates is observed. While leakage subsides with therapy, the regressed subretinal lesions are replaced by scleral staining due to destruction of the RPE, choriocapillaris, and choroid [17].

Macular leakage is another angiographic finding detected in 32% of eyes, with the extent of the leakage positively correlated with the degree of retinal disorganisation on OCT [17]. Other angiographic findings associated with VRL include periphlebitis and optic disc edema, both of which are attributed to direct lymphomatous infiltration, but nonetheless closely resemble inflammatory changes seen in uveitis [22].

Lymphomatous infiltrates produce several distinct angiographic patterns (Figure 6C). The most common is late-phase hyperfluorescent spots corresponding to pigment mottling in the mid-periphery, with a reported frequency of 93% of eyes with VRL [22]. Macular granularity is another pattern, visualised as alternating zones of hypo- and hyperfluorescence during the early and middle phases of the angiogram, with a reported frequency of 36% of eyes [17]. The leopard spot appearance can also be seen on FA, with a reported frequency of 43%–59% [19, 42].

Hyperfluorescence due to RPE atrophy is also a common FA finding in eyes with VRL, with two main patterns described: window defects and scleral staining (Figure 3C) [17, 22]. Window defects are visualised as hyperfluorescent spots with no late leakage and have a reported frequency of 86% [22] Scleral staining, reported in 55% of eyes with VRL, is visualised as late hyperfluorescence with sharp borders that correspond to early and late hypofluorescence on ICGA, hyperautofluorescence on FAF, and white lesions on fundus photography, indicating loss of both choriocapillaris and RPE [17].

#### 2.4.2 | Indocyanine Green Angiography

Indocyanine green angiography (ICGA) may have limited diagnostic utility in VRL. In a series of 43 eyes with PVRL assessed using UWF multimodal imaging, ICGA failed to identify additional lesions beyond those detected by FA and FAF and generally revealed fewer abnormalities [17]. This is in contrast with the higher yield of ICGA in inflammatory uveitides, such as white dot syndromes or sarcoidosis, where ICGA often demonstrates more abnormalities than FA [17].

Hypofluorescent lesions are a common ICGA finding in eyes with VRL (Figure 3C) [17, 22]. Two patterns have been described: small focal hypofluorescent lesions and larger confluent lesions, with reported frequencies of 77% and 31%, respectively (Figure 6D) [22]. These hypofluorescent lesions presumably result from sub-RPE lesions blocking choroidal fluorescence, are most prominent in the early phases of ICGA, and tend to diminish in later phases of ICGA [22]. In a series of 14 eyes with VRL that underwent multimodal imaging, small early-phase hypofluorescent lesions on ICGA were correlated with PEDs on OCT, hyperautofluorescent lesions on FAF, hypofluorescent lesions on FA, and small creamy lesions on fundus photography (Figure 6A) [22].

### 2.5 | Optical Coherence Tomography Angiography for Vitreoretinal Lymphoma

OCT angiography (OCTA) allows for the noninvasive visualisation of retinal and choroidal vasculature without the need for intravenous dye injection [44, 45]. When compared with conventional OCTA, swept-source OCTA (SS-OCTA) extends the imaging field beyond the macula, allowing for the detection of subtle peripheral retinal changes and capturing of extramacular VRL lesions [46].

In a series of 35 eyes with biopsy-proven VRL that underwent en face OCTA imaging, perivascular flower-bud-like lesions (PFBLs) were identified, and 34.3% of eyes had this characteristic finding [46]. PFBLs, visualised as discrete dots and confluent bands on en face OCTA, surrounded both retinal arteries and veins and created a pattern resembling ‘flower buds on frosted branches’ [46]. Despite having a similar presentation to VHRLs on OCT B-scans, they remain distinct, as PFBLs displayed a broader range of cross-sectional morphologies, were all located surrounding vessels on OCTA, and were detectable on en face OCTA, OCT B-scans, and occasionally FA and fundus photographs, not just OCT B-scans [46]. Clinically, most PFBLs appeared within 6 months from symptom onset and persisted for at least 3months before their resolution [46].

### 2.6 | Ultrasound for Vitreoretinal Lymphoma

B-scan ultrasonography may be less critical in the evaluation of most cases of VRL, as ultrasonographic findings are nonspecific and can overlap with those seen in infectious and non-infectious uveitis.

Posterior vitreous detachment is common in both VRL and uveitis [47]. Vitreoretinal adhesion, visualised as the focal convergence between the retina and the hyaloid membrane, may be seen more often in uveitis than VRL (23% vs. 6% in one series) [47]. Retinal thickening or occupying lesions can be seen as focal masses with equivalent reflectivity or diffuse thickening within the retina, which may be more common in VRL than uveitis (20% vs. 1%) [47]. Exudative retinal detachment is uncommon (10% in VRL vs. 5% in uveitis) [47].

In one’s series of 106 eyes with VRL, hyperreflective masses corresponding to vitreous infiltration were more often located in the anterior half of the vitreous, though this localization did not distinguish VRL from uveitis [47]. Centrifugal condensation of vitreous infiltration, visualised as a relatively hyporeflective central vitreous with centrifugally positioned hyperreflective opacities adjacent to the posterior vitreous cortex, was a suggestive sign for VRL, reported in 46% of eyes with VRL vs. 15% with uveitis [47].

In a separate series of 26 eyes with VRL evaluated using ophthalmic ultrasonography and UWF fundus photography, moderate to hyperreflective corpuscular material and vitreous foci were detected by ultrasonography in eyes with vitreous media opacities on UWF imaging [18]. However, this pattern was nonspecific and could not reliably distinguish VRL from infectious or non-infectious uveitis [18]. It was notable, however, that the extent of corpuscular material was correlated with the severity of vitreous media opacity [18].

## 3 | Choroidal Lymphoma

Choroidal lymphoma (CL) arises in the posterior uveal tissue following the infiltration of neoplastic lymphocytes [48]. Choroidal lymphomas are predominantly non-Hodgkin lymphomas of B-cell origin, with extranodal marginal zone B-cell lymphoma as the most common histologic subtype [1, 49–51]. Both primary and secondary forms exist, with primary CL usually presenting as a low-grade lymphoma characterised by a late onset and slow progression, whereas secondary CL presents with relatively quicker progression as a high-grade lymphoma [48, 50–52]. Patients with CL often face delays in diagnosis, up to 2years in one report [48, 51, 53]. To help prompt early referral, advancements in multimodal imaging techniques have proved useful to assist in early detection and monitoring of CL. Herein, we describe key multimodal imaging features that can help diagnose CL.

### 3.1 | Fundus Photography in Choroidal Lymphoma

**FIGURE 7 |:**
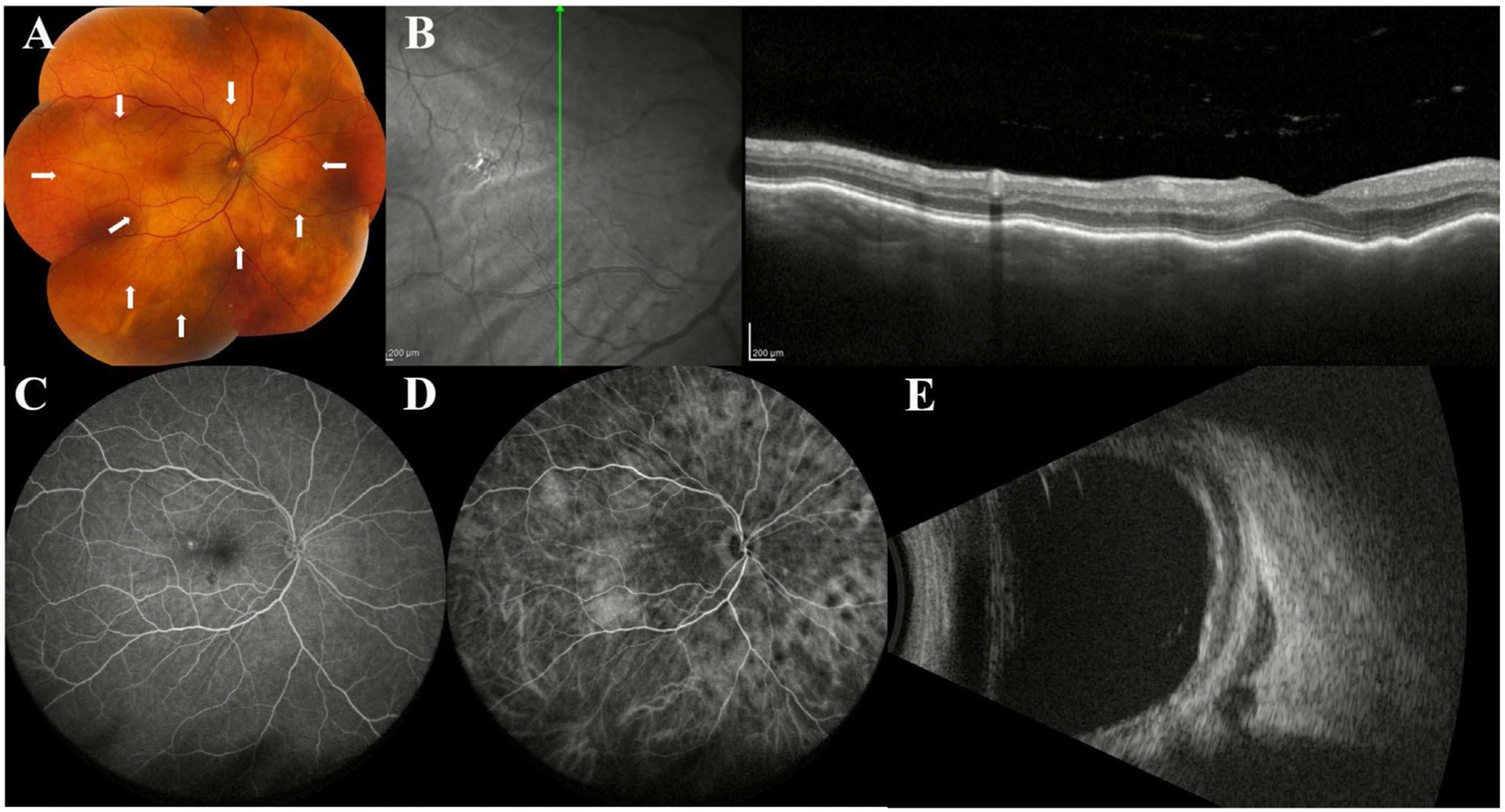
Imaging findings of choroidal lymphoma. (A) Choroidal lesions visualised on colour fundus photographs in a patient with choroidal lymphoma. (B) On OCT, choroidal lymphoma can present with a thickened and undulating appearance of the choroid, sometimes referred to as a seasick pattern. (C) Fluorescein and (D) indocyanine green (ICG) angiography can highlight hypofluorescent foci corresponding to deep choroidal lesions, more apparent on ICG. (E) B-scan may show characteristic hollow choroidal thickening, occasionally with an accompanying epibulbar mass.

Several distinct features of CL on fundus photography have been described, with choroidal infiltration being a ubiquitous finding (Figure 7A). In a series of 34 eyes of 22 patients with uveal lymphoma (21 patients with CL and 1 patient with ciliochoroidal lymphoma), yellow-white choroidal infiltrates were the dominant finding on fundus photography, detected in 100% of eyes [51]. The majority of these infiltrates were discrete, while some were placoid, with reported frequencies of 91.2% and 5.9%, respectively [51]. Diffuse choroidal thickening was another feature detected in 47.1% of eyes, and choroidal folds were detected with a frequency of 11.8% [51]. Notably, these features were most commonly located anterior to the arcades (67.6% of eyes), followed by the posterior pole (52.9% of eyes), and along the arcades (44.1% of eyes) [51]. Additionally, these features were more frequently located diffusely in all quadrants (32.4% of eyes) or in the superotemporal quadrant (32.4% of eyes) [51].

In a separate series of 6 eyes with primary CL, yellowish orange nummular infiltration was detected in all 6 eyes, and granular pigment deposits were detected in the posterior pole of 66.7% of eyes [54]. Other findings that may be detected on fundus photography in patients with CL are exudative retinal detachments, elevated mass lesions, choroidal neovascularization, optic disc swelling, and venous stasis retinopathy [53, 54].

### 3.2 | Optical Coherence Tomography in Choroidal Lymphoma

Enhanced depth imaging OCT (EDI-OCT) allows the characterisation of choroidal infiltration in CL based on lesion focality, surface contour, and infiltration depth, thus facilitating the differentiation of CL from other pathologies.

In a series of 14 eyes with CL assessed with EDI-OCT, choroidal infiltrates were classified based on focality and the displayed topography of the anterior tumour surface [55]. Three primary patterns of focality were described: unifocal, visualised as a singular choroidal lesion (frequency of 21%); multifocal, visualised as multiple nodular elevations (29%); and diffuse, visualised as the uvea entirely thickened with no clear margins (50%) [55].

Additionally, three distinct choroidal inner surface configurations may be observed: flat (calm), mini-wavy (rippled), or maxi-wavy (undulating) (Figure 7B) [55]. The wavy patterns may be described as a seasick configuration [56]. Increasing fluctuation of these topographical configurations correlates with tumour thickness on ultrasonography [48, 55], with the calm pattern corresponding to thinner lesions (mean 1.7 mm) and the undulating pattern corresponding to thicker lesions (mean 4.1 mm) [55]. The calm configuration predominated in unifocal and multifocal lesions (67% and 100% of lesions, respectively), whereas the undulating surface was most frequently associated with diffuse infiltration (71% of lesions) [55]. While these patterns may be distinctive and correlate with tumour size, they are not pathognomonic [48].

A subsequent series of 18 eyes with CL expanded on these observations by introducing choroidal infiltration depth as an additional OCT variable, categorised as deep vs. combined superficial and deep infiltration [48]. Isolated deep choroidal infiltration was correlated with longer diagnostic delay [48]. Additional anterior surface patterns, such as the lumpy bumpy configuration and the dome configuration, were introduced, with the former correlating to concurrent superficial and deep infiltration [48]. Interestingly, the extent of choroidal infiltration on OCT was incongruent with that on ICGA, with OCT displaying wider involvement [48]. This feature is of particular importance, as several diseases in the differential for CL, including other choroidal tumours and stromal choroiditis, display agreement between OCT and ICGA findings [48].

### 3.3 | Fundus Autofluorescence in Choroidal Lymphoma

There is a paucity of published studies evaluating FAF in CL. In a single study of 17 eyes with CL assessed using FAF, three distinct patterns of autofluorescence were identified: patchy, diffuse, and normal [48]. The patchy pattern, also labelled as the leopard spot pattern, was detected in 30% of eyes and was associated with increased choroidal thickness compared to eyes with normal FAF [48]. The diffuse pattern was detected in 11% of eyes [48]. A normal FAF appearance was the predominant presentation, reported in 59% of eyes [48].

### 3.4 | Fluorescein and Indocyanine Green Angiography in Choroidal Lymphoma

Both FA and ICGA may be used for characterising disease extent and laterality. ICGA, however, is considered the preferred form of angiography for CL, due to a superiority in visualising choroidal circulation [51].

FA may show early hyperfluorescence, choroidal folds, and hypofluorescent spots corresponding to choroidal infiltrates (Figure 7C) [51]. A distinct tiger-like pattern, visualised as heterogenous hyperfluorescence accompanied by RPE alterations or migrations, has been described [53]. Additional FA features such as CME, vascular staining, and disc staining are nonspecific for CL [48].

Small multifocal hyperfluorescent lesions on ICGA are a common but nonspecific feature reported in 100% of eyes in several cohorts [48, 51]. These lesions are almost always hypofluorescent in the early phase of the scan, with some becoming isofluorescent in the late phase [48]. Hypofluorescent lesions on ICGA typically correspond to yellow-white choroidal infiltrates observed on fundus examination, but they may be more apparent on ICGA than on the colour image (Figure 7D) [48, 51]. Notably, there were no associations found between these lesions and OCT pattern, choroidal thickness, or diagnostic delay [48].

### 3.5 | Ultrasound for Choroidal Lymphoma

B-scan ultrasonography is a valuable, non-invasive imaging modality for assessing CL. The classic ultrasonographic appearance of CL is smooth, diffuse, hypoechoic choroidal thickening, sometimes accompanied by hypoechoic posterior epibulbar extension at a scleral emissarial canal (Figure 7E) [52, 55]. It is notable, however, that diffuse choroidal melanoma with extraocular extension can present similarly [52].

Several patterns of choroidal infiltration have been described, including patchy, confluent, mixed, or focal-mass-like, with patchy lesions tending to be thinner and associated with better BCVA [52]. Extrascleral extension (ESE) can be detected using ultrasonography, visualised as crescentic thickening just posterior to the sclera or as discrete nodular masses, with reported frequencies of 86.4% and 45.5%, respectively, in cases of ESE [52]. In a series of 29 eyes with CL assessed using B-scan ultrasonography, ultrasound detected ESE in approximately 76% of eyes and sometimes revealed clinically unsuspected involvement of the fellow eye [52].

Ultrasonography is also useful in monitoring treatment response by allowing measurement of tumour thickness [55]. When compared with EDI-OCT, however, ultrasonography overestimated tumour thickness by 66% [55]. This discrepancy has been attributed to factors such as inaccurate ultrasonographic delineation of the choroidal-scleral interface, unintentional inclusion of tissue, and calibration incongruity [55].

## 4 | Conclusion

Diagnosing VRL and CL remains challenging due to their nonspecific clinical presentations that mimic other benign and malignant ocular diseases. While cytopathology remains necessary for definitive diagnosis, multimodal imaging plays a critical role in raising clinical suspicion, prompting early referral and diagnosis, and improving patient outcomes. Additionally, findings on multimodal imaging can help monitor disease progression and offer prognostic insights that inform clinical management.

## Figures and Tables

**TABLE 1 |:** Multimodal imaging features in intraocular lymphoma and reported frequencies.

Imaging modality	Vitreoretinal lymphoma	Choroidal lymphoma
Fundus photography	•Vitreous cellular infiltration (55%–100%) ◦ Aurora borealis (34%–34.6%) ◦ String of pearls (2%–7.7%) ◦ Nonspecific pattern (38.5%–64%) • Retinal infiltration • Subretinal infiltration (81%) • Leopard spot pattern (9%–21%)	• Choroidal infiltration (100%) ◦ Discrete choroidal infiltrates (91.2%) ◦ Placoid choroidal infiltrates (5.9%) ◦ Nummular infiltration (100%) • Diffuse choroidal thickening (47.1%) • Choroidal folds (11.8%) • Granular pigment deposits (66.7%)
Optical coherence tomography	**Vitreous** • Vitreous cell (65%–100%) • Preretinal deposits (13%–44%) **Retina** *Intraretinal infiltration* (7%–55.6%) • Diffuse intraretinal deposits (5%) • Focal intraretinal deposits (9%) • Focal round lesions • Vertical hyperreflective lesions (40%–58.3%) • Pseudonecrotic retinal lesions (7.9%–35.8%) *Outer retina* • Outer retina fuzzy border (45.5%–73.7%) • Outer retinal atrophy (10.5%–22.1%) • Ellipsoid zone disruption (52.3%–73.3%) • Hyperreflective retinal dots (72.2%) • Cloudy vitelliform submaculopathy (6%–9%) *Other retinal features* • CME (5.6%–17%) *Subretinal space* • Subretinal infiltration (53.1%–62.2%) ◦ Hyperreflective discrete nodules (21.9%–51.1%) ◦ Hyperreflective confluent bands (11.1%–31.3%) • Hyperreflective subretinal dots (45.5%) • Subretinal fluid (3.6%–27.2%) *RPE* • RPE undulation (15%–26%) • RPE nodularity (63%) • PED (25.2%–50%) *Sub-RPE* • Sub-RPE lesions (9.4%–100%)	• Choroidal infiltrates (100%) ◦ Unifocal (21%) ◦ Multifocal (29%) ◦ Diffuse (50%) • Choroidal inner surface topographical configuration ◦ Flat (calm) (50%) ◦ Mini-wavy (rippled) (14%) ◦ Maxi-wavy (undulating) (36%) ◦ Lumpy bumpy ◦ Dome
Fundus autofluorescence	Granular pattern (69%) Leopard spot appearance (34%–56%) Hyperautofluorescent lesions (100%) Blockage by mass lesion (11%)	• Patchy (leopard spot) pattern (30%) • Diffuse pattern (11%) • Normal pattern (59%)
Fluorescein angiography	• Diffuse vascular leakage (77%–100%) • Macular leakage (25%–32%) • Subretinal lesions (59%) • Macular granularity (36%) • Leopard spot appearance (43%–59%) • Late-phase hypofluorescent spots (93%) • Window defects (86%) • Scleral staining (55%)	• Early hyperfluorescence • Choroidal folds • Hypofluorescent spots • Tiger-like pattern • CME • Vascular staining • Disc staining
Indocyanine green angiography	• Small focal hypofluorescent lesions (77%) • Large confluent hypofluorescent lesions (31%)	• Small multifocal hypofluorescent lesions (100%)
Optical coherence tomography angiography	• Perivascular flower-bud-like lesions (34.3%)	• Limited information, no established pattern that contributes to diagnosis
Ultrasound	• Posterior vitreous detachment (89%) • Vitreoretinal adhesion (6%) • Retinal thickening (20%) • Exudative retinal detachment (10%) • Centrifugal condensation of vitreous media opacity (46%)	• Smooth, diffuse, hypoechoic choroidal thickening • Hypoechoic posterior epibulbar extension • Patchy, confluent, mixed, or focal-mass-like choroidal infiltration • Extrascleral extension ◦ Crescentic thickening (86.4%) ◦ Discrete nodular masses (45.5%)

Abbreviations: CME, cystoid macular oedema; PED, pigment epithelial detachment; RPE, retinal pigment epithelium.

**TABLE 2 |:** Frequency of characteristic features in vitreoretinal lymphoma versus uveitis.

OCT findings	VRL (%)	Uveitis (%)
Vitreous		
Vitreous cell or debris	48.4–93.3	54.7–67.5
Preretinal deposits	31.6–44.4^a^	7.5–9.3
Epiretinal membrane	44.2	82.6
Retina		
Central macular thickening	51.6	95.3
CME	11.7	36.0^a^
Intraretinal infiltration	34.0–53.3^a^	3.5–20.0
Vertical hyperreflective lesions	40.0^a^	20.0
Inner retinal hyperreflective spots	15.8	0.0
Outer retinal atrophy	22.1^a^	2.3
Subretinal space		
Subretinal lesions—focal	21.1–51.0	4.7–45.0
Subretinal lesions—banded	11.1–17.9	9.3–10.0
Subfoveal fluid	3.2	16.3^a^
Subretinal fluid	6.4	16.3^a^
RPE		
RPE thickening or RPE irregularity	37.9–40.0	0.0–32.5
RPE rippling (diffuse RPE elevations)	16.8–55.6	0.0–7.5
Focal RPE elevations	20.0	72.5
Sub-RPE		
Sub-RPE infiltration (deposits)	34.7–80.0	0.0–80.0
Sub-RPE focal deposits	20.0	0.0
Sub-RPE diffuse minimally-elevated deposits	18.9	0.0
Sub-RPE confluent deposits (confluent RPE detachment)	13.7–26.7^a^	0.0
Choroid		
Inner choroidal infiltration	44.4	97.5^a^

Abbreviations: CME, cystoid macular edema; OCT, optical coherence tomography; RPE, retinal pigment epithelium; VRL, vitreoretinal lymphoma.

a Key diagnostic feature.
